# Thermodynamic Insights into the Separation of Carotenoids in Reversed-Phase Liquid Chromatography

**DOI:** 10.1155/2019/7535813

**Published:** 2019-01-03

**Authors:** Nicola Marchetti, Pier Paolo Giovannini, Martina Catani, Luisa Pasti, Alberto Cavazzini

**Affiliations:** Department of Chemistry and Pharmaceutical Sciences, University of Ferrara, Italy

## Abstract

The retention mechanism of four major carotenoids, two xanthophylls (i.e., lutein and zeaxanthin) and two carotenes (i.e., lycopene and *β*-carotene), was investigated in reversed-phase liquid chromatography with the aim of thermodynamic analysis. The experimental variables considered in this study were the composition of mobile phase (MP) and the temperature. Chromatographic elutions were undertaken under linear, isocratic conditions by using a C18 stationary phase, four different MP compositions (by varying the ratio methanol/acetonitrile from 66.5/28.5 to 47.5/47.5 v/v), and column temperatures in the range 283–313 K. Traditional Van't Hoff analysis has been used to estimate changes of standard enthalpy (Δ*H*°) and Gibbs free energy (Δ*G*°) associated with the solute transfer from the mobile to the stationary phase at each mobile phase composition. The thermodynamic quantities have been correlated to the structure of investigated carotenoids and their interaction with the octadecyl silica stationary phase.

## 1. Introduction

Commons carotenoids in food are linear C_40_ polyenes formed by eight isoprene units, also called tetraterpenes, differing from each other for one or both end-groups and possibly for their cis-trans conformation. Carotenoids are primarily divided into two groups according to the presence or not of oxygen atoms: hydrocarbon carotenoids are called carotenes, while those containing oxygen are termed xanthophylls [1]. Oxygen atoms can be contained as hydroxyl, methoxy, carboxyl, keto, or epoxy groups. They are also widely distributed among plants, animals, and bacteria, serving for light harvesting and photoprotection in photosynthesis and as antioxidants [1]. Because of their importance as quality markers in food due to their medical and biological implications, carotenoids were the object of several publications in Separation Science. A large number of articles can be found in literature aimed at developing and validating HPLC and LC/MS assays for identification and determination of these molecules in food and biological matrices [2–4]. Fundamental investigations were made in the past to understand the effect of mobile phase modifiers on selectivity and resolution [5, 6], as well as the dependence of chromatographic behavior from the temperature [7, 8]. Additionally, at that time special alkyl stationary phases, such as C30, were developed and studied for improving HPLC separation of carotenoids and their isomers [9–11].

**Figure 1:**
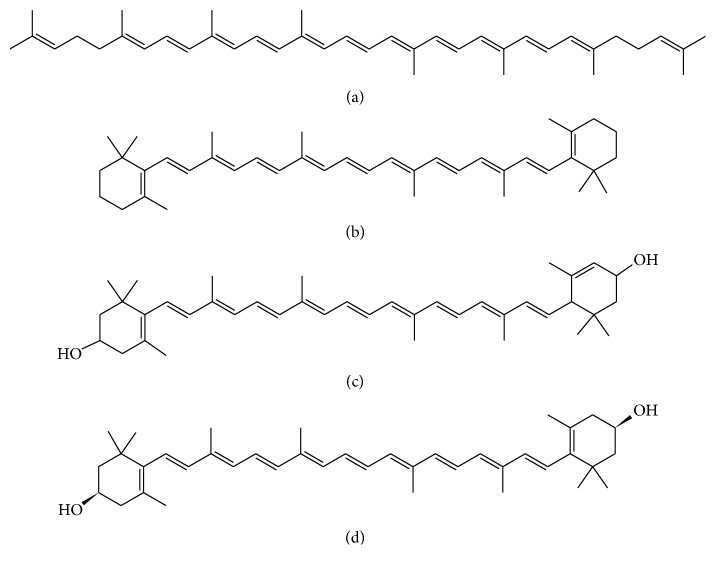
Chemical structure of (a) lycopene (LYC), (b) *β*-carotene (BCAR), (c) lutein (LUT), and (d) zeaxanthin (ZEAX).

The four major carotenoids in terms of their abundance in foods are lutein, zeaxanthin, *β*-carotene, and lycopene (see Figure 1). Lutein and zeaxanthin belong to the xanthophylls group, while lycopene and *β*-carotene are hydrocarbon carotenoids. Lycopene has two identical linear 2,6-dimethyl-1,5-heptadiene end-groups, in contrast to *β*-carotene where the same atoms are arranged into 2,6,6-trimethyl-1-cyclohexene moieties. The two xanthophylls show instead hydroxylated cyclohexene end-groups: two 4-hydroxy-2,6,6-trimethyl-1-cyclohexene for zeaxanthin, while for lutein one end-group is as before and the other is a 4-hydroxy-2,6,6-trimethyl-2-cyclohexene.

The purpose of this work is to investigate the relationships between reversed-phase chromatographic behavior of these compounds and their structural characteristics by considering the thermodynamics of retention process. In other words, the object of this study is the investigation of changes in Gibbs free energy and its contributions (i.e., variations in entropy and enthalpy) associated with the solute transfer from the mobile to the stationary phase. This have been obtained through Van't Hoff analysis by measuring the HPLC retention for the four compounds at different column temperature. After development of Van't Hoff theory, this thermodynamic analysis has been widely applied in a large number of literature works in chromatography to investigate different separation systems [12–14].

## 2. Theory

In linear conditions, the retention factor (*k*) is traditionally used to express how much longer a sample component is retarded by the stationary phase than it would take to travel through the column with the velocity of the mobile phase (MP) [15]. *k* is defined as

(1) $$\begin{array}{c} k=\frac{V_{r}-V_{0}}{V_{0}} \end{array}$$

where *V*_*r*_ and *V*_0_ are the retention and hold-up volumes, respectively. Since in linear chromatography, the distribution constant *K* (partition coefficient) is independent of sample component concentration; then *k* is also equal to the ratio of the amount of a sample component in stationary and MPs at equilibrium. Thus,

(2) $$\begin{array}{c} k=KF \end{array}$$

with *F* being the phase ratio (that is the ratio of *V*_0_ and the stationary phase volumes). During the elution process, a solute molecule continuously transfers from the mobile onto the stationary phase. The partition equilibrium constant is related to the standard free energy change, Δ*G*°, associated with solute transfer from the mobile to the stationary phase by

(3) $$\begin{array}{c} K=e^{-\Delta G^{\circ }/RT} \end{array}$$

By explicating the enthalpic and entropic contributions (Δ*H*° and Δ*S*°) to Δ*G*° and combining (2) and (3), one obtains in logarithmic form

(4) $$\begin{array}{c} \ln k=-\frac{\Delta H^{\circ }}{RT}+\frac{\Delta S^{\circ }}{R}+\ln F \end{array}$$

which shows that if Δ*H*°, Δ*S*°, and *F* are assumed to be independent of temperature (T), ln⁡*k* versus 1/*T* plots (Van't Hoff plots) are linear and allow for the estimation of Δ*H*° and Δ*S*°, provided that the phase ratio is known [16]. The measured thermodynamic quantities associated with retention represent average values due to the heterogeneity of the chromatographic surface. Yet, retention data may yield linear Van't Hoff plots, provided that the pertinent surface, solute, and solvent properties are T invariant. Solute molecules in this investigation are expected not to change with T (in the T range considered). Analogously, the density of nonpolar bonded chains of the stationary phase, their accessibility to solutes, and thus the contact surface upon binding remain T invariant.

## 3. Experimental

### 3.1. Instrumentation

The liquid chromatograph used in this work for the Van't Hoff analysis was a modular Agilent 1100 (Agilent Technologies, Santa Clara, CA). The HPLC was equipped with a solvent delivery system, a degasser, a binary pump with a static mixer, a thermostated column compartment, and a multiple wavelength diode array detector (13 *μ*L flow cell). Data acquisition frequency was 0.5 Hz.

A HPLC-MS system (Thermo Scientific) composed by a micro HPLC Surveyor Plus (pump, column thermostated compartment, autosampler, and solvent delivery system) and a LTQ XL mass spectrometer was employed for the excess isotherm measurements.

### 3.2. Column

A Bakerbond 4.6 × 250 mm column packed with 5 *μ*m C_18_ bonded silica gel particles (Mallinckrodt Baker, Phillipsburg, NJ) was used for all measurements. Column hold-up volume (measured through pycnometry; see the following) was 2.60 mL, and this well agrees with value obtained from excess isotherm measurements (i.e., 2.54 mL).

Data for Van't Hoff analysis were collected within the range 10-40° C at 277, 287, 291, 295, 298, 303, 308, and 313 ±0.1 K. Temperatures were controlled by a digital thermometer placed inside the column compartment.

### 3.3. Chemicals

Acetonitrile (ACN), methanol (MeOH), dichloromethane (DCM), butylated hydroxytoluene (BHT), triethylamine (TEA), ammonium acetate, lutein (LUT), zeaxanthin (ZEAX), lycopene (LYC), and *β*-carotene (BCAR) were purchased by Sigma (Sigma-Aldrich, St. Louis, MO).

### 3.4. Mobile Phases

MPs were prepared by mixing two nonaqueous solutions at three different proportions, channel-A/channel-B 70:30, 60:40, and 50:50, through the binary HPLC pump. Channel-A delivered a 0.05 M ammonium acetate, 0.1% (w/v) BHT, and 0.05% (v/v) TEA solution in MeOH:DCM 95:5 (v/v). DCM as a nonpolar solvent may significantly affect the solubility and the distribution between stationary and mobile phases of sample compounds (with effects on retention and peak shape). The solution in channel-B was a 0.1% (w/v) BHT and 0.05% (v/v) TEA solution in ACN/DCM 95:5. Accordingly, the four isocratic MP compositions employed in the chromatographic measurements were MeOH:ACN:DCM 66.5:28.5:5, 57:38:5, and 47.5:47.7:5.

### 3.5. Excess Isotherm and Pycnometric Measurements

A chromatographic column can be characterized in terms of its thermodynamic and kinetic parameters (i.e., void volume, phase ratio, stationary phase volume, etc.) by means of specific measurements, such as pycnometry and determination of excess isotherms [17–20]. Here, excess isotherm of ACN from ACN/MeOH mixtures was measured to set up the experimental design in terms of ACN volume fraction to be investigated. Other studies about excess isotherms are beyond the purposes of this work. A short view of the most relevant equations employed in tracer pulse chromatography to measure excess isotherm will be presented in the Supplementing Materials [18, 21–25].

The most popular of the static methods for the determination of void volume is pycnometry (or the weight difference method). The method consists in weighing the chromatographic column sequentially filled with solvents of different densities. The pycnometric void volume, *V*_0,*pycn*_, is thus calculated as [19, 21, 22]

(5) $$\begin{array}{c} V_{0,pycn}=\frac{w_{1}-w_{2}}{\delta_{1}-\delta_{2}} \end{array}$$

where *w*_1_ and *w*_2_ are the weights of the column filled with solvent 1 and 2, respectively, and *δ*_1_ and *δ*_2_ their densities. It is generally agreed upon that *V*_0,*pycn*_ gives the maximum possible void volume.

## 4. Results and Discussion

**Figure 2:**
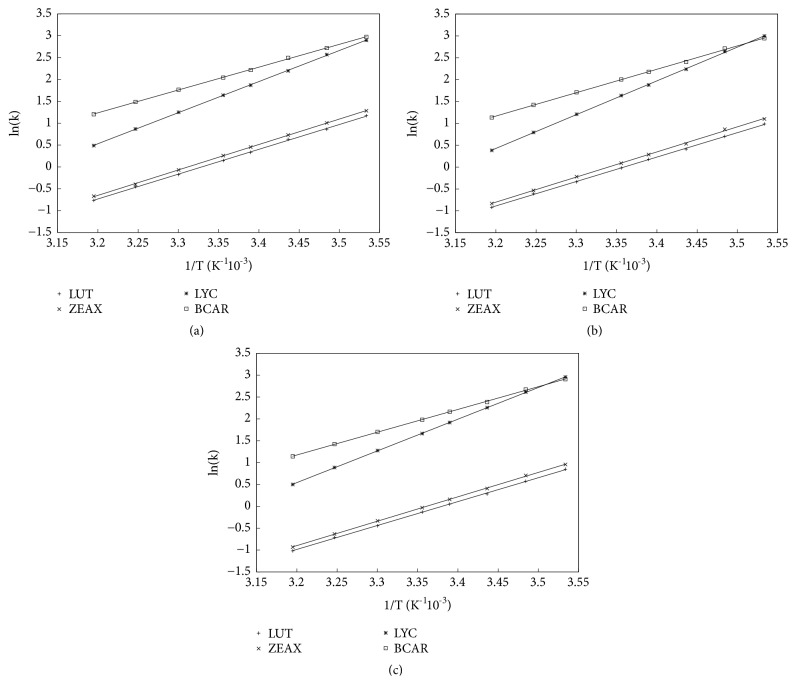
Van't Hoff plots at different MP composition: (a) ACN=28.5% v/v; (b) ACN=38% v/v; (c) ACN=47.5% v/v.

Figures 2(a), 2(b), and 2(c) report the values of ln⁡*k* measured at different temperatures for each MP composition: Figure 2(a) refers to MeOH:ACN:DCM 66.5:28.5:5; Figure 2(b) refers to MeOH:ACN:DCM 57:38:5, and Figure 2(c) refers to MeOH:ACN:DCM 47.5:47.5:5. Experimental points are well fitted to a linear first-order equation. The plots revealed some structural differences among xanthophylls (carotenoids containing oxygen atoms) and carotenes (hydrocarbon carotenoids). The straight lines, in fact, can be visually divided into two groups: the upper one for stronger retained compounds such as carotenes (LYC and BCAR) and the lower one for less retained species encompassing xanthophylls (ZEAX and LUT). Additionally, in all plots the singular behavior of LYC can be highlighted: the slope of fitted straight line for LYC totally differs from the other three. At all MP compositions by decreasing the temperature, chromatographic behavior of LYC was characterized by a larger increase of retention than BCAR. A deep look at both fitted line parameters revealed that slope and intercept for LUT and ZEAX decreased with the increase of ACN in MP; instead for LYC and BCAR they had highest slope and lowest intercept at 38% ACN, while for smaller or larger values (28.5% and 47.5%, respectively) they were roughly the same. Changes in fit parameters were considerably smaller for BCAR and this led to a general behavior of carotenoid retention (i.e., ln⁡(k)) as a function of ACN in MP where only LYC displayed a U-shaped trend within the investigated range, whereas LUT, ZEAX, and BCAR decreased their retention with the increase of ACN. As the main consequence, the chromatographic resolution (i.e., the ratio between retention factors) between LUT/ZEAX and LYC/BCAR behaved differently with variation of ACN in MP: in particular, no significative change for the two xanthophylls was observed, while for the two carotenes resolution decreased by almost 9% when ACN increases.

Retention data used to estimate thermodynamic quantities through Van't Hoff analysis revealed that BCAR was always more retained than LYC at all MP compositions and standard column temperatures. Only for MPs ACN/MeOH/DCM 38/57/5 and 47.5/47.5/5 (v/v) it happened that, decreasing T, a loss of resolution between LYC and BCAR can be firstly observed, and then an inversion of the elution order of these compounds occurred.

Since solubility of xanthophylls was found not to significantly change in the range of employed MP compositions, the increase in retention, associated with the decrease of ACN in the MP, might be explained by the competitive effect of the MeOH molecules. MeOH is potentially able to compete with xanthophylls (containing OH moieties) on residual silanols present on octadecylsilane derivatized silica surfaces. Due to their hydrophobic nature (see Figure 1), carotenes were significantly more retained than xanthophylls. Solubility of carotenes, contrary to xanthophylls, was significantly reduced in moderately polar MPs (that is, by increasing MeOH percentage in MP) and this could explain the observed increase in retention.

**Figure 3:**
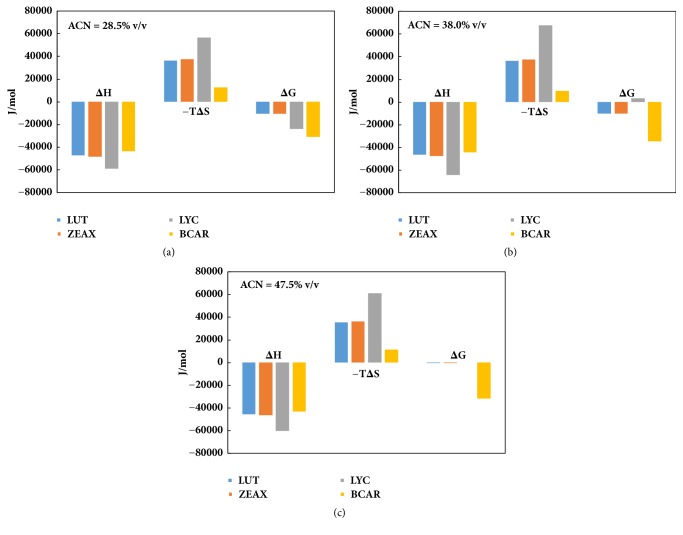
Thermodynamic quantities estimated by Van't Hoff analysis at 298 K at different MP composition: (a) ACN=28.5% v/v; (b) ACN=38% v/v; (c) ACN=47.5% v/v.

For better investigating the chromatographic behavior of carotenoids, the enthalpic and entropic contribution to the solute transfer from the mobile to the stationary phase was estimated by means of the traditional Van't Hoff analysis (4). Figures 2(a)–2(c) show the plots of ln⁡*k* as function of the inverse of T for the MP compositions employed in this work. For all carotenoids, Δ*H*° and Δ*S*° have negative values. Their effect on retention is thus opposite: a negative Δ*H*° causes an increase in *k*, while a negative Δ*S*° lowers it (see (4)). Thus, we can expect a certain enthalpy-entropy compensation and this can be discussed also in terms of resulting Gibbs free energy Δ*G*°. Firstly, one can observe that for LYC, Δ*H*° and *T*Δ*S*° had the largest absolute values between all carotenoids, regardless of the MP composition. On the opposite side, for BCAR a larger difference between Δ*H*° and *T*Δ*S*° can be noted and this ended up with the most negative values of Δ*G*° at all MPs. For LUT and ZEAX the enthalpy-entropy compensation was more marked than that for the two carotenes. Particularly, when ACN=47.5% (v/v), Δ*G*° was very close to 0, and this happened also for BCAR (Figure 3(c)).

Statistical significance of our data was evaluated by descriptive statistics associated with calculation performed: relative standard deviation for regression of Van't Hoff data points was always lower than 1%. Additionally, a multiple comparisons t-test was performed with critical values at 95% of probability for evaluation of statistical differences between changes of thermodynamic quantities. This is the reason why error bars were not reported, as they were meaningless for the discussion of results.

Our findings can be correlated with the molecular structure of xanthophylls and carotenes (see Figure 1). In particular, they reflected the tendency of weakly polar substances to expel, in the adsorption process, polar molecules (i.e., organic solvent) and to establish interactions with other nonpolar species (i.e., the octadecyl chains on the stationary phase) in order to maintain a favorable environment.

The significantly large Δ*H*° observed for LYC can be explained by the molecular end-groups (i.e., linear) of similar polarity and interactive characteristics with stationary phase which all contribute to a favorable change in the enthalpy associated with the phase transfer. The same did not happen with the other carotenoids considered in this work. The presence of rings (i.e., BCAR) and/or OH moieties (i.e., LUT and ZEAX) on the structures led to a less favorable Δ*H*° in the transfer from the mobile to the stationary phase. From this analysis it followed also that the linear structure of LYC is also responsible for the largest observed term *T*Δ*S*°. The phase transfer of LYC occurred together with a relevant loss of degrees of freedom for this molecule. In the adsorbed state, the molecule assumed a privileged position in which the overlap between its alkyl moieties and the alkyl chains on the stationary phase is maximum. Additionally, a complete viewpoint of this retention mechanism had to take into account the stationary phase composition (i.e., excess isotherm, see Figure S1 in Supplementing Materials). The MP compositions investigated were chosen on the basis of ACN excess isotherm: the maximum of ACN concentration on the stationary phase was estimated to take place around 38% v/v, while the other two ACN contents were selected just before (i.e., 28.5% v/v) and after (i.e., 47.5% v/v) this maximum. In fact absolute values of Δ*H*° and *T*Δ*S*° are smaller in Figures 3(a) and 3(c), while the largest values arose for ACN=38% v/v (Figure 3(b))

As it happened for LUT and ZEAX, polarity of BCAR was not very different from that of LYC and the same occurred about their solubility. However, the reason for their differences in chromatographic retention can be ascribed to the presence of two end rings on the molecular structure of BCAR: these end-groups profoundly affect the arrangement of molecules on the stationary phase. This was demonstrated by the fact that the measured Δ*H*° for LYC had the most negative value (i.e., see Figures 3(a)–3(c)). BCAR had Δ*H*° value very close to those for LUT and ZEAX. In order to fully understand the retention model, the terms *T*Δ*S*° had to be discussed as well. It was interesting to observe that more polar molecules, such as LUT and ZEAX (i.e., containing OH moieties), were provided for more negative *T*Δ*S*° values than BCAR (i.e., enthalpy-entropy compensation). This ended up with smaller absolute Δ*G*° than BCAR and this meant, obviously, lower chromatographic retention. Conversely, LYC had the largest loss in degrees of freedom (i.e., increasing order) upon the transfer from the mobile to the stationary phase. In two cases (see Figures 3(b) and 3(c)) this produced a larger enthalpy-entropy compensation than that for LUT and ZEAX, while only at ACN=28.5% (Figure 3(a)) LYC had a slightly less negative Δ*G*° value than BCAR. A possible reason for this described thermodynamic behavior of LUT and ZEAX can be the additional entropy decrease originated by the onset of hydrogen bond interactions between OH moieties and the residual silanols on the stationary phase surface: the role of hydroxyl groups on xanthophylls and the way they interact with the stationary phase well correlate with the variation of Δ*H*° and *T*Δ*S*° for LUT and ZEAX with the amount of MeOH in MP. Methanol can act as a competitor for LUT and ZEAX and this is the reason why the mobile phase composition was another important variable to consider. Although a given effect on thermodynamic quantities by the relative amount of MeOH in the mobile phase can be expected, if Figures 3(a)–3(c) are compared, only an extremely small effect on LUT, ZEAX, and BCAR will be observed. Since the relative amounts of ACN in the MP investigated were across the maximum ACN absorption, these MP compositions produced little or even no effects on Δ*H*° and *T*Δ*S*°. For LYC the variations of Δ*H*° and *T*Δ*S*° were more complex. When taken individually, enthalpy and entropy changes varied with excess isotherm for acetonitrile: maximum (absolute) values at ACN=38% and lower (absolute) quantities at ACN=28.5% and 47.5%. The absolute value of Δ*G*° decreased with the increase of ACN: when ACN=38%, Δ*G*° became slightly positive; when ACN=47.5%, Δ*G*° is essentially equal to zero (i.e., enthalpy/entropy compensation). This effect can be advocated as one possible explanation as evidenced in Figure S3 (Supplementing Materials). By plotting Δ*H*° versus *T*Δ*S*°, linearity of data can be noticed in the case of LUT, ZEAX, and LYC and, hence, for these compounds an enthalpy/entropy compensation seems a reasonable effect that influences the final Δ*G*° values. Additionally, the same plot shows no enthalpy/entropy compensation, or at least on a different level from the other three compounds.

## 5. Conclusions

The traditional Van't Hoff analysis applied to the study of carotenoids has evidenced that, at least for the four investigated ones, there is a strict correlation between their molecular structures and the chromatographic behavior on a C18 column. Due to the presence or absence in their structures of OH moieties, xanthophylls (LUT and ZEAX) or carotenes (LYC and BCAR) exhibit completely different features and chromatographic behaviors that are explicable on the basis of the observed enthalpic and entropic contribution to Gibbs free energy in the solute transfer from the mobile to the stationary phase. It is worth pointing out that conclusions regarding the influence of structural features on retention are based on the behavior of just four compounds, so that generalization to other carotenoids cannot be taken for granted.

In particular, the linearity of the structure of LYC is responsible for the largest (as absolute values) changes in both adsorption entropy and enthalpy. By changing the temperature it was possible to invert the elution order between LYC and BCAR. Thermodynamically, octadecyl silica is not sufficiently selective to separate LUT and ZEAX, since interactions with OH moieties do not occur directly on the C18 chains, but perhaps are secondary, unspecific interactions that exist because of free silanols on the silica surface.

## Data Availability

(1) The chromatographic data used to support the findings of this study are included within the article and within the supplementary information file. (2) The chromatographic raw and elaborated data used to support the findings of this study are available from the corresponding author upon request.

## Supplementary Materials

Supplementary Materials This part includes a brief description of excess isotherm theoretical background and how it can be experimentally measured. Figure S1) shows the measured excess isotherm of acetonitrile on the stationary phase from the mobile phase. Figure S2 reports an example of HPLC separation between the four carotenoids, object of this study. Then, Figure S3 includes data measured by Van't Hoff analysis (i.e., Δ*H*° and *T*Δ*S*°) to show experimental evidence of enthalpy-entropy compensation for lutein, zeaxanthin, and lycopene, but not for *β*-carotene.
