# Supplementary material for: Thermodynamic Insights into the Separation of Carotenoids in Reversed-Phase Liquid Chromatography
Source: Int J Anal Chem. 2019 Jan 3;2019:7535813. doi: 10.1155/2019/7535813 (PMC6335859; doi:10.1155/2019/7535813)
Supplement: Supplementary Materials — This part includes a brief description of excess isotherm theoretical background and how it can be experimentally measured. Figure S1) shows the measured excess isotherm of acetonitrile on the stationary phase from the mobile phase. Figure S2 reports an example of HPLC separation between the four carotenoids, object of this study. Then, Figure S3 includes data measured by Van't Hoff analysis (i.e., ΔH° and TΔS°) to show experimental evidence of enthalpy-entropy compensation for lutein, zeaxanthin, and lycopene, but not for β-carotene. [file 7535813.f1.pdf]

Thermodynamic insights into the separation of carotenoids in reversed-phase liquid chromatography

Nicola Marchetti*, Pier Paolo Giovannini, Martina Catani, Luisa Pasti, Alberto Cavazzini

Department of Chemistry and Pharmaceutical Sciences, University of Ferrara, Italy

Supplementing Material

Tracer Pulse Chromatography

According to the tracer pulse (TP) method for a binary system, operational definitions of the excess volume of the isotopically labeled i compound, V_i^{exc}, and of the thermodynamic void volume, V_0, are:

$$V_i^{exc} = (V_{R,i}^* - V_{R,j}^*)\theta_i^M \theta_j^M \tag{1}$$

and

$$V_0 = V_{R,i}^* \theta_i^M + V_{R,j}^* \theta_j^M \tag{2}$$

where $V_{R,i}^*$ and $V_{R,j}^*$ are the elution volumes for each labeled component i and j, and θ_i^M and θ_j^M are the volume fractions in the bulk mobile phase. Excess adsorption isotherms can be employed to estimate the capacity and thickness of the surface phase (absolute adsorption). The volume of i in the stationary phase, V_i^S, and the stationary phase volume, V_S, can be assessed by the linear region of the excess isotherm, being

$$V_i^{exc} = V_i^S - V_S \theta_i^M \tag{3}$$

According to eq.2, V_0 is obtained by injecting small samples of eluent with isotopically labeled solute components and determining the elution volumes (i.e., $V_{R,i}^*$ and $V_{R,j}^*$) for each labeled component. Obviously, the method can be used with a pure eluent (i.e., θ_i^M or $\theta_j^M = 1$) and this is by far the simplest way of determining V_0.

*Corresponding author: nicola.marchetti@unife.it (N. Marchetti)

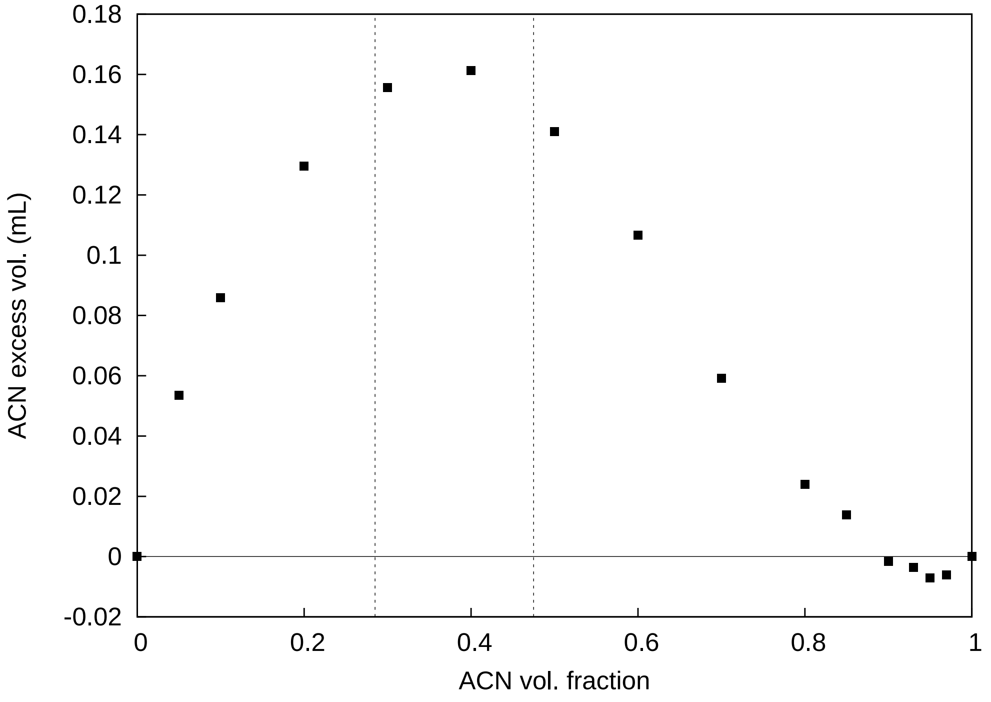

Figure S1. Acetonitrile excess isotherm. Vertical dashed lines indicate the region within ACN volume fraction are investigated.

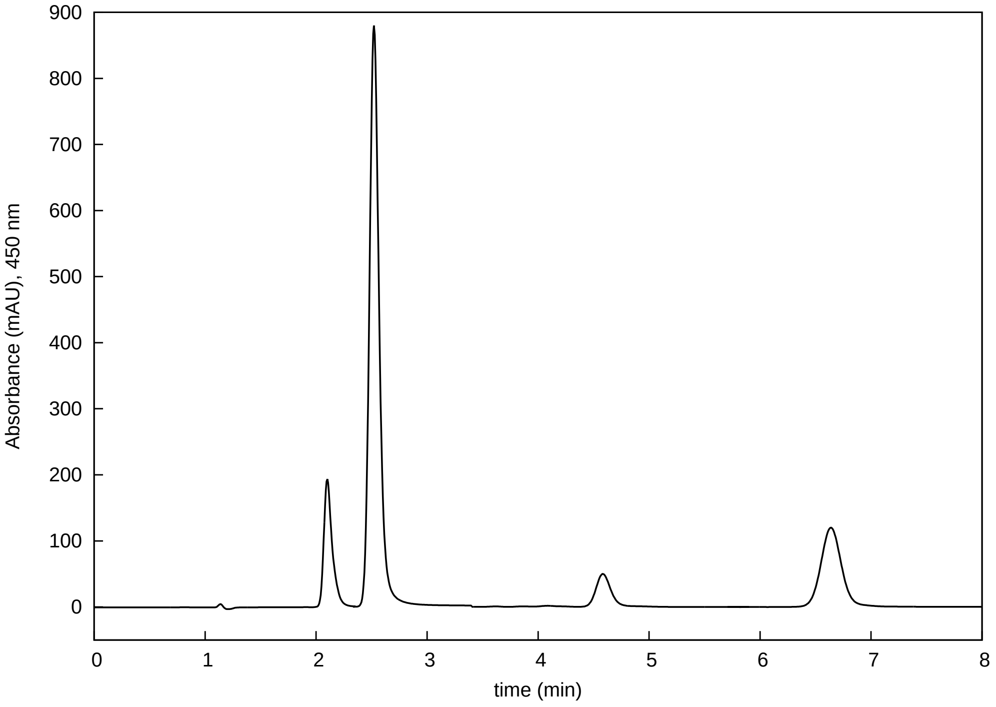

Figure S2. HPLC separation between lutein, zeaxanthin, lycopene and β-carotene. Cromatographic conditions were: column temeprature 30°C; MeOH:ACN:DCM 57:38:5.

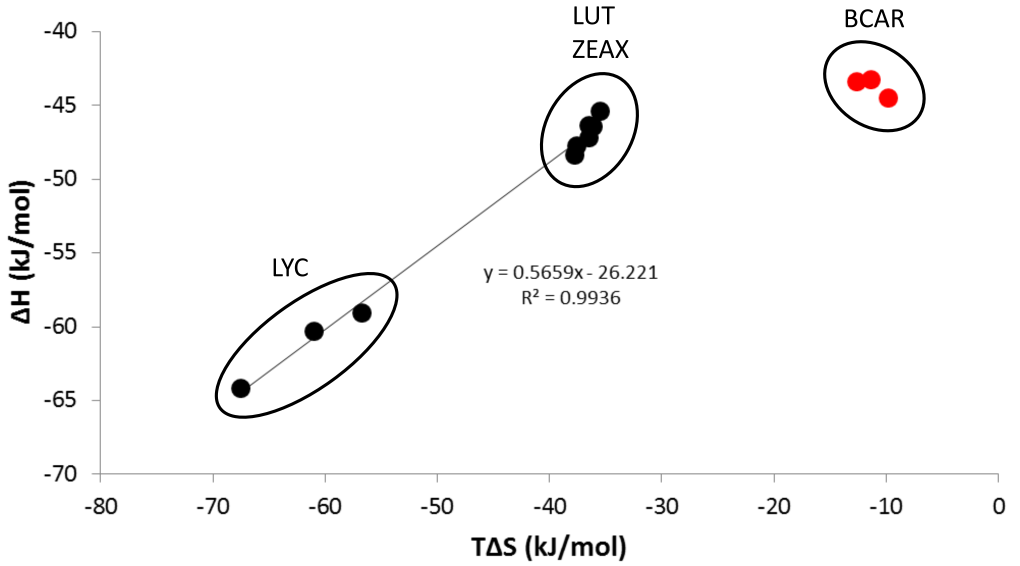

Figure S3. Evidence of enthalphy-entropy compensation for lutein, zeaxanthin and lycopene.
